# Case Report: Complete remission in a neonate with high-risk neuroblastoma harboring MYCN amplification and 1p deletion: a case for aggressive early intervention, and literature review

**DOI:** 10.3389/fped.2025.1641407

**Published:** 2026-01-14

**Authors:** N. Kh. Gabitova, I. N. Cherezova, I. V. Osipova, D. I. Sadykova, Dalal Nasr, Ayman A. Gobarah, Ahmed Arafat

**Affiliations:** 1 Pediatrics Department, Kazan State Medical University, Kazan, Russia; 2 Pediatric Department of the State Autonomous Heath Institution, Children’s Republican Clinical Hospital (CPCH), Kazan, Russia; 3 Pediatrics Department, Kids Heart Medical Center Abu Dhabi, Abu Dahbi, United Arab Emirates; 4 Pediatric Department Faculty of Medicine, Suez Canal University, Ismailia, Egypt; 5 Pediatrics Department, Dubai Academic Health Corporation, Dubai, United Arab Emirates; 6 Pediatrics Department, Jiahui International Hospital, Shanghai, China

**Keywords:** neuroblastoma, neonate, high-risk, MYCN amplification, hematopoietic stem cell transplantation

## Abstract

Neuroblastoma is the most prevalent extracranial solid tumor in infancy and early childhood, accounting for 8%–10% of all pediatric malignancies and contributing significantly to cancer-related mortality. Its clinical spectrum ranges from spontaneous regression to aggressive metastatic disease, often influenced by underlying genetic aberrations such as MYCN amplification and chromosomal deletions (1p, 11q, and 17q). We present a rare case of a full-term male neonate diagnosed with stage 4 neuroblastoma originating from the left adrenal gland, exhibiting both MYCN amplification and 1p deletion. The patient had extensive liver metastases and supradiaphragmatic lymphadenopathy at diagnosis. Multimodal treatment, including intensive chemotherapy per the NB2004 protocol, surgical resection, high-dose consolidation chemotherapy, and autologous hematopoietic stem cell transplantation (AHSCT), led to complete remission by 11 months of age. Despite severe post-transplant complications such as sepsis and enteropathy, the patient remained disease-free with normal developmental milestones at follow-up. To the best of our knowledge, this is the first reported case of neonatal neuroblastoma with concurrent MYCN amplification and 1p deletion achieving favorable outcome through comprehensive multimodal therapy. This case underscores the importance of early diagnosis, genetic profiling, and aggressive treatment in managing high-risk neuroblastoma in neonates.

## Introduction

Neuroblastoma (NB) is an embryonal malignancy derived from neural crest cells of the developing sympathetic nervous system. It most frequently arises in the adrenal medulla or in the paraspinal sympathetic ganglia. Neuroblastoma occurs exclusively in children; the formation of the sympathetic ganglia is completed by approximately five years of age (1–3). It is the most common malignancy in infants (age <1 year), with a median age of diagnosis of approximately 18 months. Neuroblastoma accounts for about 8%–10% of all pediatric cancers (including leukemias) and is the third leading cause of childhood cancer mortality (1, 2, 4, 5). The etiology of neuroblastoma remains unknown; epidemiological studies have not established definitive associations with environmental exposures, parental occupational factors, prenatal medication use, maternal smoking, or prenatal alcohol consumption (1–3). Neuroblastoma is initiated by somatic mutations in neural crest–derived embryonic neuroblasts, which fail to differentiate into mature sympathetic ganglion cells or adrenal medullary cells and instead continue to proliferate unchecked. Such genetic alterations are thought to occur *in utero*. The vast majority of neuroblastoma cases are sporadic; familial cases are rare (≤1.2% of patients) and have been reported in association with hereditary cancer predisposition syndromes such as neurofibromatosis type 1, Sotos syndrome, Weaver syndrome, Costello syndrome, Noonan syndrome, and Beckwith–Wiedemann syndrome (6). Genetic aberrations play a central role in neuroblastoma pathogenesis: for example, amplification of the MYCN oncogene (present in ∼20%–30% of cases) and deletion of the chromosome 1p region (particularly at 1p36) are among the most significant markers of unfavorable prognosis and aggressive tumor behavior (6–10). MYCN amplification is associated with rapid tumor progression, treatment resistance, and high relapse rates, and it frequently co-occurs with 1p36 deletions (8–10). Other recurrent genomic alterations include deletion of chromosome arm 11q and gain of the long arm of chromosome 17 (17q). Neuroblastoma exhibits a wide spectrum of clinical behavior, ranging from spontaneous regression or maturation into a benign ganglioneuroma to widespread metastatic disease (1, 11). Approximately 40%–50% of patients present with metastatic disease at diagnosis, with common sites of spread including the bone marrow, bones, lymph nodes, liver, and skin (3, 10, 11). Clinical manifestations depend on the location and extent of disease: tumors in the adrenal or retroperitoneal regions may present with abdominal pain or neurologic deficits due to spinal cord compression, whereas thoracic tumors may cause respiratory symptoms or ipsilateral Horner syndrome. Systemic or metastatic features can include hepatomegaly, bone pain, subcutaneous nodules, mucosal bleeding, and pancytopenia (10–12). Pain is reported in approximately 35% of patients (8). Diagnosis involves a combination of imaging and pathological evaluation: conventional radiography, ultrasound, computed tomography (CT), magnetic resonance imaging (MRI), and multi-slice CT (MSCT) are used to detect the primary tumor, while functional imaging (e.g., ^123^I-MIBG scintigraphy) and bone marrow biopsy are used to assess metastatic spread. Definitive diagnosis requires histopathological and immunohistochemical analysis of tumor specimens, and molecular genetic studies (particularly assessment of MYCN amplification and 1p36 deletion) provide important prognostic information (9). Serum neuron-specific enolase (NSE) is a useful tumor marker in neuroblastoma; elevated levels correlate with increased tumor burden and a worse prognosis, whereas lower levels are associated with better outcomes. Treatment decisions are guided by risk stratification schemes that incorporate patient age, disease stage (e.g., INSS/INRG stage), tumor histology, MYCN status, and genetic abnormalities such as 1p36 deletion (10). We report a rare case of neonatal stage 4 neuroblastoma with MYCN amplification and 1p deletion, presenting with extensive metastases. Aggressive multimodal therapy, including AHSCT, achieved complete remission by 11 months of age. Written informed consent was obtained from the parents of the infant for the publication of any potentially identifiable images or data included in this article.

## Case presentation

A male infant with uneventful perinatal period, born at 41–42 weeks gestation (birth weight: 3,700 g, length: 54 cm) to a primigravida with a history of threatened preterm labor, iron-deficiency anemia, and retinal angiopathy. Apgar scores: 8–9. Early adaptation was uncomplicated, with exclusive breastfeeding. At 15 days of age, he presented with irritability, abdominal distention, and hepatomegaly (liver palpable below the umbilicus). Ultrasound revealed a large abdominal mass, hepatosplenomegaly, and ascites. Urgent hospitalization to the Children's Republican Clinical Hospital (Kazan) followed.

### Patient perspective and psychosocial context

Written informed consent for publication was obtained from the parents. They provided a brief perspective on their experience, stating: “The decision to proceed with such intensive treatment for our newborn was the most difficult of our lives. We relied completely on the expertise and clear communication of the medical team. The journey was emotionally devastating, but seeing our son now, healthy and reaching his milestones, makes every challenging moment worth it.” The family history was non-contributory for neuroblastoma or other cancer predisposition syndromes. The psychosocial burden was significant, requiring the family to relocate temporarily to be near the tertiary care center.

On examination, pallor, abdominal circumference 45 cm, hepatosplenomegaly, prominent abdominal vasculature. Laboratory findings included: moderate anemia (RBC 2.62 × 10^12^/L, Hb 86 g/L, Hct 26.2%), thrombocytopenia (Plt 34 × 10⁹/L), elevated alkaline phosphatase (359 U/L), AST (124.2 U/L), AFP (6,267 IU/mL), and NSE (>200 µg/L). Bone marrow aspirate showed no tumor cells.

**Figure 1:**
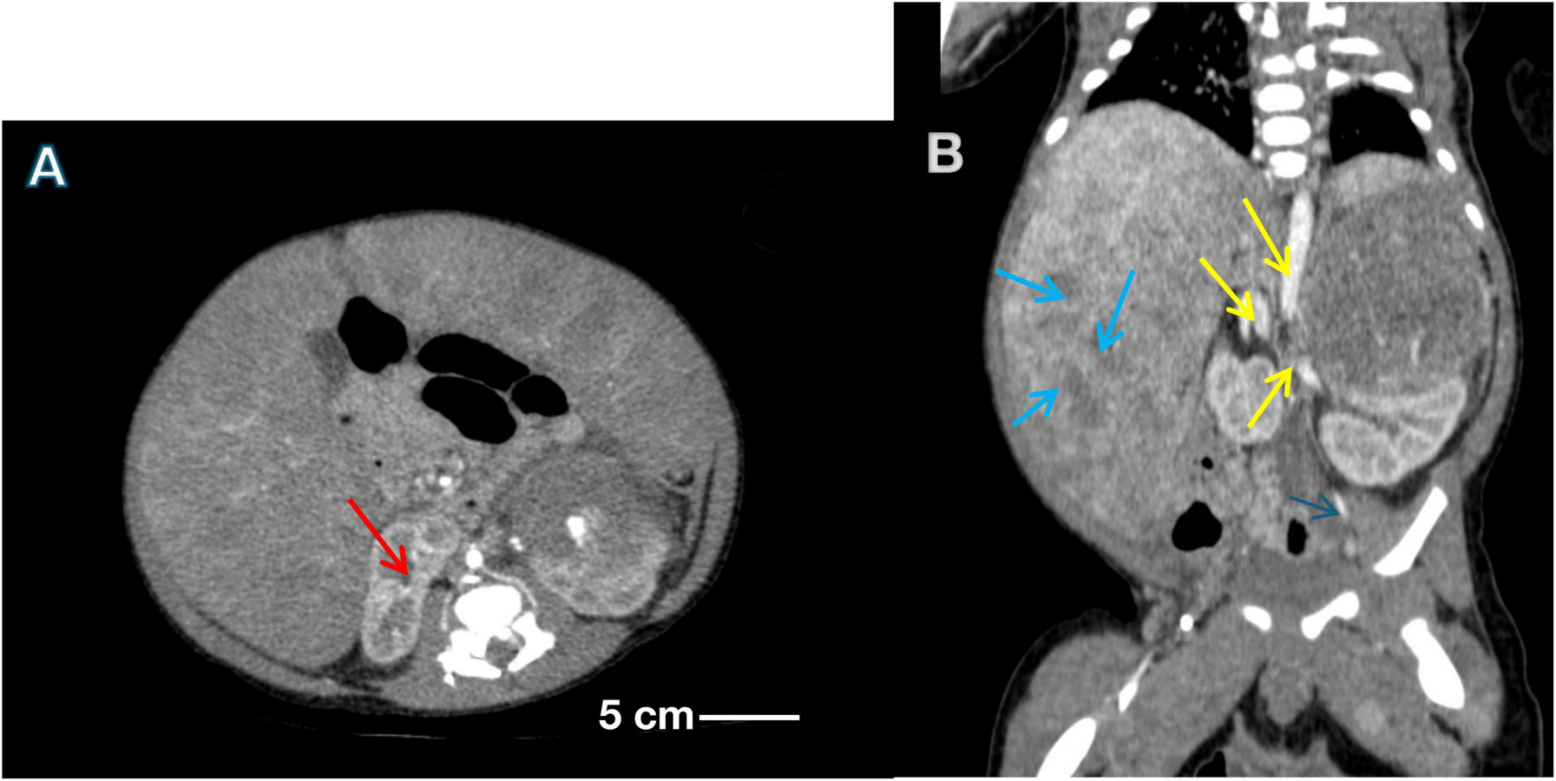
**(A)** Contrast-enhanced CT scan of the abdomen showing a large, lobulated, heterogeneous mass (red arrows) arising from the left adrenal region, displacing the kidney inferiorly and medially. **(B)** Axial CT section demonstrating multiple hypodense hepatic nodules (blue arrows) consistent with liver metastases, and enlarged para-aortic lymph nodes (yellow arrows) indicating regional lymphadenopathy. Scale bar = 5 cm. Findings correspond to Stage 4 disease (INSS classification). The diagnosis of high-risk neuroblastoma was confirmed histologically and by molecular testing showing MYCN amplification and 1p36 deletion, both prognostically adverse markers that guided aggressive multimodal therapy.

Abdominal ultrasound/CT showed large heterogeneous left adrenal mass (55 × 57 × 55 mm) displacing the left kidney; multiple hepatic/splenic nodules; retroperitoneal lymphadenopathy; ascites. (Figures 1A,B), thoracic CT revealed signs of pulmonary hypertension, right bronchial narrowing, diaphragmatic metastases, and enlarged lymph nodes.

### Diagnostic challenges

The initial presentation of a neonate with hepatomegaly, abdominal distension, and ascites raised a broad differential diagnosis, including other embryonal tumors (e.g., hepatoblastoma), congenital hepatic fibrosis, and metabolic storage diseases. The rapid identification of a large left adrenal mass on ultrasound, however, quickly narrowed the diagnostic focus to neuroblastoma.

An ultrasound-guided biopsy of the adrenal mass was performed, and histopathology demonstrated poorly differentiated neuroblastoma with positive staining for synaptophysin, chromogranin, and NB84. Histological examination of the tumor biopsy yielded a conclusion: poorly differentiated neuroblastoma with a low index of mitotic count. Fluorescence *in situ* hybridization (FISH) and comparative genomic hybridization (CGH) confirmed MYCN amplification and 1p36 deletion. Based on the International Neuroblastoma Risk Group (INRG) classification, the patient was designated high-risk. According to the International neuroblastoma staging system (INSS, 1986), there are four clinical stages, and on the basis of the studies performed, the child was given a clinical diagnosis: low-grade neuroblastoma of the left adrenal gland, stage 4, metastatic liver disease, supradiaphragmatic lymph nodes. High risk group.

By day 5, the infant developed respiratory distress, hemodynamic instability, worsening anemia (Hb 60 g/L), thrombocytopenia (Plt 18 × 10⁹/L), and cyanosis. Mechanical ventilation and inotropic support were initiated. Polychemotherapy (NB2004 protocol) commenced at 1 month. After two cycles, tumor and liver size decreased, NSE declined to 18.9 µg/L. Post-5 cycles, the adrenal mass (43 × 50 × 50 mm) persisted.

At this stage, the patient's abdominal circumference had increased to 54 cm, accompanied by the development of lower extremity edema extending to the hips, significant scrotal swelling with marked tension, and urinary dysfunction. Due to the critical nature of the patient's condition, definitive surgical excision of the tumor was postponed until clinical stabilization could be achieved. During the intensive care unit (ICU) stay, the patient was managed with inotropic support, hemostatic agents, corticosteroid therapy, transfusional support with packed red blood cells and fresh frozen plasma, as well as broad-spectrum antimicrobial therapy.

### Treatment and surgical intervention

At one month of age, the patient commenced polychemotherapy per the high-risk NB2004 protocol, with dosing adjusted for neonatal physiology. The regimen consisted of alternating cycles of vincristine (0.025 mg/kg/day), cyclophosphamide (300 mg/m^2^), doxorubicin (15 mg/m^2^), cisplatin (90 mg/m^2^), and etoposide (200 mg/m^2^) (13). A detailed summary of the treatment schedule and dosing is provided in Table 1.

**Table 1:** Summary of multimodal treatment and clinical course.

Treatment phase	Timing (age)	Intervention	Key agents & dosing	Response/outcome
Presentation & diagnosis	15 days	Clinical evaluation, imaging, biopsy	–	Stage 4 neuroblastoma, MYCN amp, 1p del, extensive metastases
Initial stabilization	15–30 days	ICU management	Mechanical ventilation, inotropic support, transfusions	Stabilization of respiratory and hemodynamic status
Induction chemotherapy	1–5 months	NB2004 Protocol (5 cycles)	Cycle A: Vincristine (0.025 mg/kg), Cyclophosphamide (300 mg/m^2^), Doxorubicin (15 mg/m^2^) Cycle B: Cisplatin (90 mg/m^2^), Etoposide (200 mg/m^2^)	Marked reduction in tumor burden, NSE normalized (18.9 µg/L), resolution of respiratory distress
Surgical resection	5 months	Left adrenalectomy	–	Gross total resection; histology showed >90% necrosis
Consolidation & AHSCT	7 months	High-dose chemotherapy with autologous stem cell rescue	Conditioning: Carboplatin (6.6 mg/kg), Etoposide (5 mg/kg)	Complicated by sepsis, enteropathy, myocarditis; resolved with intensive care
Maintenance therapy	8–17 months	Isotretinoin	13-cis-retinoic acid (80 mg/day, approx. 160 mg/m^2^) for 9 months	Completed without major toxicity
Follow-up	20 months	Clinical, imaging, tumor markers	–	Asymptomatic, no evidence of disease, normal development

Clinical response was rapid, with marked resolution of pain, edema, and respiratory distress observed after two cycles. By treatment day 20, serum neuron-specific enolase (NSE) had normalized to 18.9 µg/L, accompanied by significant reductions in tumor and liver volumes. Follow-up thoracic CT confirmed the absence of pulmonary metastases, lymphadenopathy, or airway compromise. However, imaging identified a persistent left adrenal mass (43 × 50 × 50 mm) with deformation of the upper pole of the left kidney, alongside persistent hepatosplenomegaly with multiple parenchymal lesions.

Following five cycles of induction chemotherapy, and based on sustained normalization of NSE, significant radiographic tumor reduction, and clinical stabilization, the patient underwent surgical resection of the left adrenal tumor at five months of age. Histopathological assessment of the specimen revealed extensive therapy-induced changes, characterized by widespread tumor necrosis and calcifications, confirming a robust response to neoadjuvant chemotherapy.

Post-operative recovery was followed by high-dose consolidation chemotherapy with carboplatin (6.6 mg/kg) and etoposide (5 mg/kg), culminating in autologous hematopoietic stem cell transplantation at seven months of age.

### Post-transplant and maintenance therapy

At seven months of age, the patient underwent autologous hematopoietic stem cell transplantation at a specialized pediatric oncology center.

The early post-transplantation period was complicated by sepsis, gastrointestinal enteropathy, and myocarditis. Following recovery from these acute complications, the patient commenced maintenance therapy with 13-cis-retinoic acid (Isotretinoin) at a dose of 80 mg/day (approx. 160 mg/m^2^) for a total of 9 months. Immunotherapy with anti-GD2 antibodies was not administered due to its unavailability on the national pharmaceutical market at the time of treatment.

### Current Status

At the most recent follow-up, at 11 months of age, the patient was asymptomatic and demonstrated normal growth and development, with a body weight of 10,300 g.

Abdominal and retroperitoneal ultrasonography did not reveal any evidence of residual or recurrent tumor.

The serum neuron-specific enolase (NSE) level was 21.1 pg/mL. Routine hematologic and biochemical laboratory values remained within normal age-appropriate limits.

## Discussion

High-risk neuroblastoma in neonates is an exceptionally rare entity, and its management represents one of the most formidable challenges in pediatric oncology. This case is particularly unique due to the patient's age at presentation, the coexistence of two significant genetic aberrations (MYCN amplification and 1p deletion), and the extensive disease burden at diagnosis. The ability to achieve complete remission despite such a poor initial prognostic profile highlights significant advances in diagnostic and therapeutic modalities.

Early detection in this case was facilitated by clinical vigilance and the judicious use of imaging and laboratory diagnostics. The prompt histopathological and molecular analysis allowed for precise risk stratification, enabling initiation of an appropriately aggressive treatment plan.

Several studies underscore the relevance of MYCN amplification in predicting adverse outcomes (11, 12, 14). Its presence is linked to rapid tumor progression, metastasis, and resistance to conventional therapies. Likewise, 1p chromosomal deletion is independently associated with poor event-free and overall survival (12, 15). The co-occurrence of both markers further diminishes survival chances, necessitating multimodal, high-intensity treatment.

The patient's treatment adhered to the NB2004 protocol, a widely accepted regimen that emphasizes early induction therapy followed by surgical resection, high-dose consolidation chemotherapy, and AHSCT (16). Surgical timing is crucial—performed after tumor burden is reduced but before fibrosis complicates resection. In this case, histological findings of massive necrosis and calcification following induction chemotherapy validated the decision to proceed with surgery at five months.

Postoperative consolidation with high-dose chemotherapy followed by AHSCT remains a cornerstone in treating high-risk neuroblastoma. Several studies have demonstrated improved long-term survival in patients who undergo myeloablative therapy and stem cell rescue (9, 17). Despite the risks associated with transplantation, including infection, mucositis, and organ toxicity, the benefits in carefully selected cases outweigh the complications, as demonstrated here.

Moreover, the case draws attention to the need for supportive care infrastructure, including neonatal intensive care units equipped to manage the complications of aggressive cancer therapy. Early multidisciplinary involvement—including pediatric oncologists, surgeons, intensivists, geneticists, and pathologists—proved essential in the successful outcome.

From a research perspective, this case adds to the relatively limited body of literature on neonatal neuroblastoma with high-risk features. Most studies focus on older infants and children, while reports on neonates remain scarce. The favorable outcome observed here, despite high-risk genetic markers and extensive disease, suggests that even neonates may benefit significantly from aggressive, protocolized treatment regimens.

Additionally, advances in genomics and personalized medicine offer exciting avenues for future treatment. The integration of molecular markers such as ALK mutations, segmental chromosomal aberrations, and expression profiling may help further stratify risk and refine therapeutic approaches (18, 19). This case reinforces the potential of combining conventional therapy with targeted approaches, immunotherapy, and precision diagnostics.

Recent work of literature highlight the potential of artificial intelligence in enhancing the diagnostic evaluation of neuroblastoma. In particular, Ramesh et al. demonstrated that AI-based image analysis of standard hematoxylin and eosin–stained slides can reliably classify tumor morphology and predict MYCN amplification. These findings support the integration of AI-driven tools into pediatric oncology to improve diagnostic accuracy and risk stratification in neuroblastoma management (20).

The achievement of complete remission in a neonate with both MYCN amplification and 1p deletion is exceptionally rare and represents a significant challenge in pediatric oncology. The co-occurrence of these two genetic markers typically confers a dismal prognosis, with rapid progression and resistance to therapy often observed even in older infants (8, 9, 12). To contextualize the outcome of our patient, we reviewed the sparse literature on neonatal neuroblastoma with MYCN amplification (Supplementary Table S1). This comparison underscores the aggressiveness of this disease entity in the earliest period of life.

For instance, Alhazmi & Bamehrez (2024) reported a neonate with MYCN-amplified stage 4S disease who succumbed within three months despite therapeutic intervention (5). In a larger cohort study, Li et al. (2024) highlighted that the combination of MYCN amplification with segmental chromosomal alterations like 1p deletion was associated with particularly poor event-free survival, a finding reflected in their reported case of a 21-day-old with MYCN/1p-altered neuroblastoma who experienced progressive disease by eight months (4). Even with aggressive treatment including AHSCT, outcomes remain guarded, as illustrated by a case from Matthay et al. (1999) where a 14-day-old infant with MYCN-amplified stage 4 disease died from treatment-related toxicity shortly after transplantation (9).

In this grim context, the sustained complete remission achieved in our patient at 20 months of age is highly notable. While rare cases of spontaneous regression in MYCN-amplified 4S disease exist (e.g., Brodeur, 2003), this phenomenon is not applicable to our case, which presented with widespread metastatic stage 4 disease requiring immediate, intensive therapy (3). The favorable outcome we observed is therefore most plausibly attributed to the rapid initiation of a multimodal, high-intensity protocol, timely surgical intervention upon achieving maximal response, and meticulous supportive care that enabled the patient to withstand the significant toxicities of treatment, including a complicated post-transplant course. This case adds to the limited but critical body of evidence suggesting that with comprehensive and protocol-driven management, a curative outcome is possible even in neonates presenting with the most high-risk genetic features.

## Strengths and limitations

This case report has several strengths, including the detailed documentation of a successful, aggressive multimodal approach in a neonate, with precise genetic and treatment data. However, it also has limitations. As a single-case report, its findings may not be generalizable. The follow-up, while now extending to 20 months, is still intermediate-term; long-term surveillance is crucial to monitor for disease recurrence and late effects of intensive therapy. Furthermore, the unavailability of anti-GD2 immunotherapy represents a deviation from the current standard of care in many countries, and its inclusion might have further improved the prognosis. Despite these limitations, this case provides a valuable template and a message of hope for managing similar challenging cases.

## Conclusion

This report documents a rare presentation of high-risk neuroblastoma in a neonate characterized by MYCN amplification and 1p deletion, with a favorable outcome following an aggressive multimodal treatment approach. The early diagnosis, rapid initiation of therapy, genetic profiling, and multidisciplinary care played pivotal roles in the patient's survival and remission.

The rarity of this case, especially the achievement of complete remission despite multiple poor prognostic factors in a neonate, underscores the evolving landscape of neuroblastoma treatment and highlights how even the most challenging cases can achieve positive outcomes with precision medicine and coordinated care.

## Data Availability

The original contributions presented in the study are included in the article/Supplementary Material, further inquiries can be directed to the corresponding author.
