# Supplementary material for: Case Report: Complete remission in a neonate with high-risk neuroblastoma harboring MYCN amplification and 1p deletion: a case for aggressive early intervention, and literature review
Source: Front Pediatr. 2026 Jan 14;13:1641407. doi: 10.3389/fped.2025.1641407 (PMC12847230; doi:10.3389/fped.2025.1641407)
Supplement: Supplementary file 1 [file Table1.docx]

Case Report: Complete remission in a neonate with high-risk neuroblastoma harboring MYCNamplification and 1p deletion: a case for aggressive early intervention, and literature review

N.Kh. Gabitova¹, I.N. Cherezova¹, I.V. Osipova², D.I. Sadykova ^1^, Dalal Nasr^3^, Ayman A. Gobarah^4,5^, Ahmed Arafat^1,6*^

^1^ Kazan State Medical University, Pediatrics Department, Kazan, Russia.

^2^ Pediatric Department of the State Autonomous Heath Institution «Children’s Republican Clinical Hospital (CPCH), Kazan, Russia.

^3^ Kids Heart Medical Center Abu Dhabi, Pediatrics Department, Abu Dhabi, UAE.

^4^ Pediatric department Faculty of Medicine Suez Canal university, Ismailia, Egypt.

^5^ Dubai Academic Health Corporation, Pediatrics Department, Dubai, United Arab Emirates

^6^ Jiahui International Hospital, Pediatrics Department, Shanghai, China.

***** Correspondence: [dr.a.zaher@hotmail.com](mailto:dr.a.zaher@hotmail.com); Tel.: +79270444210

****Supplementary Table S1: Reported Cases of Neonatal Neuroblastoma with MYCN Amplification****

| Study (Year) | Age at Diagnosis | | | Stage | MYCN Status | 1p Status | Other Genetic Features | | Treatment | Outcome | Follow-up |
| --- | --- | --- | --- | --- | --- | --- | --- | --- | --- | --- | --- |
| ****Current Case (2024)**** | | 15 days | 4 | | Amplified | Deleted | None reported | NB2004 chemo, surgery, AHSCT, isotretinoin | | Complete remission | 20 months |
| Alhazmi & Bamehrez (2024) | | 1 day | 4S | | Amplified | Not reported | - | Chemotherapy (unspecified) | | Died | 3 months |
| Khan et al. (2021) | | 28 days | 4 | | Amplified | Not reported | - | Intensive chemo, AHSCT | | Partial response | 12 months |
| Li et al. (2024) | | 21 days | 4 | | Amplified | Deleted | 11q deletion | Chemo, surgery | | Progressive disease | 8 months |
| Matthay et al. (1999) | | 14 days | 4 | | Amplified | Not reported | - | Chemo, AHSCT, isotretinoin | | Died from toxicity | 5 months |
| Brodeur (2003) | | 7 days | 4S | | Amplified | Not reported | - | Supportive care only | | Spontaneous regression | 24 months |

*Key Observations from Comparative Analysis:*

- Neonatal MYCN-amplified neuroblastoma has historically poor outcomes
- Combination MYCN amp + 1p del is particularly rare in neonates
- Aggressive multimodal therapy including AHSCT appears necessary for survival
- Spontaneous regression can occur in 4S disease even with MYCN amplification
- Treatment-related toxicity is a major concern in this population
